# Dolutegravir combined anti-retroviral agents as second-line drugs and virologic suppression of adults with HIV in Federal Teaching Hospital, Ido Ekiti - case series

**DOI:** 10.11604/pamj.2025.50.99.40350

**Published:** 2025-04-10

**Authors:** Oluwaserimi Adewumi Ajetunmobi, Wasiu Adegbenga Ajetunmobi, Olumide Emmanuel Adewara, Olusegun Emmanuel Gabriel-Alayode, Oluwafemi Johnson Adegbamigbe, Olusegun Emmanuel Omosanya, Helen Titilayo Ilori

**Affiliations:** 1 Department of Family Medicine, Afe Babalola University Ado Ekiti/Federal Teaching Hospital, Ido Ekiti, Nigeria,; 2 Department of Paediatrics, Afe Babalola University Ado Ekiti/Federal Teaching Hospital, Ido Ekiti, Nigeria,; 3 Department of Obstetrics and Gynaecology, Afe Babalola University Ado Ekiti/Federal Teaching Hospital, Ido Ekiti, Nigeria,; 4 Department of Haematology and Blood Transfusion, Afe Babalola University Ado Ekiti/Federal Teaching Hospital, Ido Ekiti, Nigeria,; 5 Department of Family Medicine, Jericho Specialist Hospital, Ibadan, Nigeria

**Keywords:** Dolutegravir, transition, antiretroviral therapy, case series

## Abstract

The transition to dolutegravir (DTG)-combined anti-retroviral therapy (ART) as a preferred option in both first- and second-line for all populations was adopted by the World Health Organization (WHO) due to encouraging safety data for women and adolescent girls during the peri-conception period. This case series aimed at determining the viral suppression of adults with HIV on Dolutegravir combined anti-retroviral agents as second-line drugs. Four clients' viral suppression were observed from ART initiation to switching to second line and later transiting to the DTG-combined regimen and the findings was that clients A and B, who were transitioned to the DTG-combined regimen had superior viral suppression by three months when compared to clients C and D who were switched to second line. The case series supports the WHO recommendation that the DTG-based regimen is the preferred first- and second-line switch ART regimen in HIV management, in achieving faster viral load suppression than any other regimen.

## Introduction

The effective management for human immunodeficiency virus (HIV) disease has been the use of anti-retroviral therapy (ART). This usually involves the combination of at least two nucleoside and/or nucleotide reverse transcriptase inhibitors (NRTIs) like Tenofovir Disoproxil Fumarate (TDF), Lamivudine (3TC), Emtricitabine (FTC) and the third agent from any of Non-Nucleoside Reverse Transcriptase Inhibitors (NNRTIs) such as Efavirenz (EFV) and Nevirapine (NVP), Protease Inhibitors (PIs) such as Lopinavir/Ritonavir (LPV/r), Atazanavir (ATV), Integrase Strand Transfer Inhibitors (INSTIs) such as Dolutegravir (DTG) and Raltegravir (RAL) [1]. Over the years, people living with HIV have benefited from ART, whether as a first-line regimen or second-line drugs, and the goal of treatment is to attain persistent HIV viral load suppression, which permits immunological recovery and reduces HIV transmission among the population. Achieving viral load suppression, therefore, is dependent on adherence to highly active anti-retroviral therapy (HAART) and the use of optimized antiretroviral (ARV) drug regimens, which significantly improve treatment outcomes in HIV-positive clients [2].

A fixed dose combination of ART with low pill burden, good admissibility, little or low toxicity, ease of monitoring, and a high resistance barrier also plays a role [3]. DTG as an INSTI has been found to offer better tolerability, efficacy, and durability than EFV when combined with TDF and 3TC [3]. The transition to dolutegravir-combined first-line regimen was supported by the WHO particularly in low and middle income countries (LMICs) and the updated consolidated HIV guidelines released by the WHO also supported adopting DTG as a preferred option in both first- and second-line ART for all populations due to encouraging safety data for women and adolescent girls during the peri-conception period [4-6]. A study conducted by Katbi *et al*. on virologic response among Key Populations (KP) living with HIV following a switch to dolutegravir-based regimen in Southern Nigeria showed that the DTG-based regimen exhibited superior virologic suppression in transitioned KP at 3-months compared to KP on TLE for 6-months [7]. This case series aimed to corroborate that transitioning to a DTG-based regimen would result in significant virological suppression in adults with HIV.

## Methods

**Study design:** this was a case series of four clients accessing care at the Federal Teaching Hospital, Ido-Ekiti (FETHI).

**Study setting:** the FETHI was founded in 2014 from formerly Federal Medical Centre, Ido-Ekiti which was established in July 1998 from the then Ido General Hospital of 1954. FETHI is a referral centre for all the health institutions (specialist hospitals, general hospitals, comprehensive health centers) in Ekiti State. The hospital runs inpatient, general, and specialized outpatient clinics with 28 departments, out of which 18 are in the clinical services area, while the rest are administrative and supportive services. The hospital has 168 beds and offers healthcare services in family medicine, internal medicine, surgery, obstetrics and gynaecology, paediatrics, psychiatry, dental, orthopaedics, ear, nose and throat (ENT), ophthalmology, community medicine, laboratory medicine, radiology and physiotherapy. The family medicine clinic runs every day of the week and manages undifferentiated diseases (irrespective of age and gender), including people living with HIV. The adult retroviral clinic of the hospital is being referred to as the care and support clinic and is run twice a week. Currently, there are over 1000 clients active on ART. The newly diagnosed clients and those previously on either TDF/3TC/EFV (TLE) based first-line had been transitioned to TLD regimen. However, some clients were switched from first-line to second-line drugs because they were virally unsuppressed.

**Study participants:** four clients, who presented to the hospital at different times, were diagnosed with HIV, commenced and monitored on ART. Two of which did not do well on first-line ART, were switched to second-line (TDF/3TC/ATV/r) and the other two clients who were also switched to second line but later transitioned to TLD with good virological suppression observed after a follow up viral load test conducted at three-months after transiting to TLD, based on the new national guideline [5] which specified three-month viral load assessment for clients on TLD.

**Data source:** this was through their case files and investigation charts.

**Variables:** the client A was referred to as CA, client B as CB, C as CC, and D as CD.

**Approval:** permission and consent to report these findings were sought from the clients despite the fact that the information concerning them was coded.

## Results

Client CA, who was a 48-year-old female Tailor, Yoruba, Christian, whose reason for hospital encounter nine years prior was one month history of diarrhoea. She was diagnosed with HIV, WHO stage II disease and commenced on AZT/3TC/NVP ART after the baseline investigation results were found to be within the normal reference limits. The T-cell (CD4) count at ART initiation was 10 cells/mm^3^. Two years later, she was switched to TDF/3TC/LPV/r when there was no viral suppression despite good medication adherence and enhanced adherence counselling. By mid-2021, she had transitioned to TLD with good virological suppression. Table 1 shows the CD4 count and viral load results.

**Table 1:** CD4 count and viral load results of client A (CA)

Dates	CD4 count (cells/mm^3^)	Dates	Viral load (copies/ml)
05/11/2013	10	19/11/2015	2,345 switched to the second line
11/02/2014	60	08/07/2017	1,445
30/07/2015	67	02/08/2018	872
19/11/2015	115	27/06/2019	903
10/03/2016	150	27/06/2020	6,350
21/07/2016	244	10/06/2021	1,889 transitioned to TLD
08/07/2017	395	08/09/2021	<20
		01/11/2021	<20
		25/07//2022	<20

The data shows the CD4 count results and viral loads of client A at the initiation of ART and at the time of switch and transition; CD4: T-cell (white blood cells that fight infection); TLD: Tenofovir Lamivudine Dolutegravir antiretroviral therapy combination

Client CB was a trader, Yoruba and a Christian, whose symptom at presentation was two months history of unexplained weight loss and who was diagnosed with HIV disease, WHO stage I at age 46 years. She was commenced on ART - AZT/3TC/NVP at initiation, which was later switched to second-line drug (TDF/3TC/LPV/r) two years later when there was no clinical improvement. She was later transitioned to TLD 09/02/2021 with good virological suppression and clinical outcome. Table 2 shows her CD4 count and viral load results.

**Table 2:** CD4 count and viral load results of client B (CB)

Dates	CD4 count (cells/mm^3^)	Dates	Viral load (copies/ml)
16/05/2015	339	24/03/2017	3,090 switched to the second line
02/04/2016	244	05/07/2018	580
24/03/2017	203	07/02/2019	655
15/03/2018	453	07/02/2020	6,790
05/07/2018	576	20/07/2020	2,523
		09/02/2021	4,162 transitioned to TLD
		08/05/2021	<20
		18/07/2022	<20

The CD4 count of CB dropped from baseline, and there was also increased viral load about two years after commencement of ART, but it dropped when she was switched to second-line ART; TLD: Tenofovir Lamivudine, Dolutegravir antiretroviral therapy combination

Client CC, now a 36-year-old P3^+ 0^3 alive Yoruba, trader and Christian, presented to the clinic on 30^th^ June, 2011 with three months history of fever and weight loss and one month history of passage of loose stools. She was diagnosed with HIV disease stage IIB, tuberculosis (TB) status 1. Her baseline CD4 count at initiation of ART was 20 cells/mm^3^. She was commenced on ZDV/3TC/NVP combination on 04/07/2011 after other preliminary results were assessed and within normal limits. In addition to her drugs, she was given 960 mg daily of Cotrimoxazole. She was switched to second-line drugs - TDF/3TC/ATV/r on account of immunological and virological failure on 04/08/2016. Her CD4 count and viral load results over the years are as shown in Table 3.

**Table 3:** CD4 count and viral load results of client C (CC)

Dates	CD4 count (cells/mm^3^)	Dates	Viral load (copies/ml)
04/07/2011	20	04/08/2016	39,356 drugs switched to second line
07/06/2012	299	27/07/2017	12, 500
02/05/2013	269	12/07/2018	6,200
09/11/2013	218	07/03/2019	1,300
15/05/2014	103	02/01/2020	939
2015	defaulted	25/11/2021	94
05/04/2016	18	16/10/2022	<20
04/08/2016	18		
27/07/2017	301		
12/07/2018	409		

The data was sourced from the client records at presentation, initiation of therapy, and at transitioning to dolutegravir (DTG)

Client CD was a 42-year-old P2^+0^2 alive woman, Yoruba by tribe, a civil servant, and a Christian who presented with a one-month history of passage of loose stools. She was later diagnosed with HIV, WHO stage II. Her baseline CD4 count was 87 cells/mm^3^. She was commenced on ART - AZT/3TC/NVP combination. She was changed to a second-line drug - TDF/3TC/ATV/r when there was no suppression. Her CD4 at drug switch was 346 cells/mm^3^, and VL was 79,903 copies/ml (Table 4).

**Table 4:** CD4 count and viral load results of client D (CD)

Dates	CD4 count (cells/mm^3^)	Dates	Viral load (copies/ml)
10/04/2011	87	13/12/2016	36,158
13/07/2012	333	22/08/2017	79,903 switched to the second line
06/01/2014	320	26/06/2018	8,460
12/08/2015	214	27/08/2019	1,115
22/08/2017	346	28/07/2020	322
01/03/2018	380	25/05/2021	146
26/06/2018	420	17/05/2022	81

The CD4 counts and viral loads were obtained from CD records

## Discussion

All four clients were females showing that females are more affected by the disease when compared with males. This was consistent with United Programme on HIV/AIDS (UNAIDS) data signifying that HIV infection is disproportionately higher among females in Nigeria [8]. Their age range was between 15-49 years at the time of diagnosis, also corroborating the UNAIDS report that women aged 15-49 years are more than twice as likely to be living with HIV as men 1.9% versus 0.9% [7]. Clients CA and CB were unable to achieve untransmittable viral load value of <200 copies/ml until they were transitioned to TLD. Clients CC and CD, on the other hand, took four and three years, respectively, to achieve untransmittable viral load value of <200 copies/ml despite being switched to the second-line regimen. Client CC defaulted at a time which could be due to her being temporarily relieved of symptoms, fed up with the daily dose regimen or being introduced to other alternatives with the false hope that she would get better clinically.

Clients A and B, who were switched to a second-line regimen before being transitioned to TLD-based regimen, had superior viral suppression by three months of TLD when compared to clients C and D, who were switched to second line. This was similar to the findings by Katbi *et al*. [7], and other studies that more participants achieved virological suppression at 3 months when they were transitioned to TLD compared to TLE and more participants on TLD also achieved un-transmittable viral load level of < 200 c/ml in three months compared to un-transmittable viral load level achieved while on TLE for a longer period of six months [9-11].

**Limitations:** some viral load results that could have indicated viral suppression or lack thereof were missing during the routine investigations in the early years of ART.

## Conclusion

The goal of the treatment of people living with HIV is to attain viral suppression, hence the commencement of first-line ART and transition to second-line in case of treatment failure. Dolutegravir-based ART was therefore introduced as a first-line regimen and was later observed to be useful when the second-line regimen did not give significant and persistent viral suppression. The case series therefore supports the recommendation by the WHO that the DTG-based regimen is the preferred ART regimen in the management of HIV in achieving viral load suppression faster than any other regimen.

### What is known about this topic

- The effective management of HIV disease involves the use of anti-retroviral therapy (ART), which is highly active;
- The WHO has approved dolutegravir (DTG)-combined ART as a preferred option for all patients with HIV due to safety data seen in women and adolescent girls during the peri-conception period;
- The transition to a DTG-based regimen would result in significant virological suppression in adults with HIV.

### What this study adds

- Dolutegravir-based ART was observed to be a useful regimen in patients who had already switched to the second-line regimen but did not have significant and persistent viral suppression;
- Transitioning such patients to dolutegravir combined ART gives a very remarkable viral suppression within three months of doing so.
